# Melatonin rescued interleukin 1β-impaired chondrogenesis of human mesenchymal stem cells

**DOI:** 10.1186/s13287-018-0892-3

**Published:** 2018-06-14

**Authors:** Bo Gao, Wenjie Gao, Zizhao Wu, Taifeng Zhou, Xianjian Qiu, Xudong Wang, Chengjie Lian, Yan Peng, Anjing Liang, Jincheng Qiu, Yuanxin Zhu, Caixia Xu, Yibing Li, Peiqiang Su, Dongsheng Huang

**Affiliations:** 1 Department of Orthopedics, Sun Yat-sen Memorial Hospital of Sun Yat-sen University, #107 West Yan Jiang Road, Guangzhou, 510120 Guangdong China; 2 Department of Orthopedics, The First Affiliated Hospital of Sun Yat-sen University, Guangzhou, Guangdong China; 3 Research Centre for Translational Medicine, The First Affiliated Hospital of Sun Yat-sen University, Guangzhou, Guangdong China; 4 Guangdong Provincial Key Laboratory of Orthopedics and Traumatology, The First Affiliated Hospital of Sun Yat-sen University, #58 Zhongshan Road II, Guangzhou, 510080 Guangdong China; 5 Department of Spine Surgery, Xi’an Honghui Hospital, Xi’an Jiaotong University, Xi’an, China

**Keywords:** Human mesenchymal stem cells, Chondrogenesis, Melatonin, Interleukin-1β, Metabolic balance, Apoptosis

## Abstract

**Background:**

Osteoarthritis (OA) is a widespread arthritic disease and a primary cause of disability. Increasing evidence suggests that inflammation has a pivotal part in its pathogenesis. Interleukin-1β (IL-1β) is a primary mediator of local inflammatory processes in OA. Current therapies for OA mainly focus on the symptoms of the advanced stage of the disease. The possible utilization of bone marrow mesenchymal stem cells (BMSCs) to regenerate cartilage is an appealing method, but in the case of OA requires chondrogenesis to take place within an inflamed environment. Our previous study showed that melatonin (MLT) can promote chondrogenic differentiation of MSCs, but whether MLT can rescue IL-1β-impaired chondrogenesis in human BMSCs has not yet been established. MLT, which can have anti-inflammatory and prochondrogenic effects, has demonstrated potential in defeating IL-1β-induced inhibition of chondrogenesis and further study should be conducted.

**Methods:**

Human bone marrow-derived MSCs were separated and cultured based on our system that was already documented. A high-density micromass culture system was used for the chondrogenic differentiation of human BMSCs, which was also described previously. Human BMSCs were induced for chondrogenesis for 7, 14, and 21 days with the treatment of IL-1β and MLT. The cultured cartilage pellets were then evaluated by morphology, extracellular matrix accumulation, and chondrogenic, metabolic, and apoptotic marker expression. Furthermore, cell apoptosis was assessed by TUNEL assay. The phosphorylation level P65 and IκBα of the NF-κB pathway activity was explored on day 21 of chondrogenic differentiation of BMSCs.

**Results:**

The current evaluation showed that MLT can save IL-1β-impaired chondrogenesis of human BMSCs in different aspects. Firstly, MLT can restore the chondrogenic pellet size, and rescue matrix synthesis and accumulation. Secondly, MLT can upregulate chondrogenic marker COL2A1 expression at both mRNA and protein levels, and also regulate the expression levels of other chondrogenic markers like *ACAN*, *SOX9*, and *COL10A1* in the presence of IL-1β. Thirdly, MLT can maintain the metabolic balance of the chondrogenic process by suppressing expression of catabolic genes, such as *MMP*, *MMP13*, and *ADAMTS4*. Furthermore, MLT can subdue IL-1β-induced cell apoptosis of BMSCs throughout chondrogenesis. Meanwhile, MLT suppressed the phosphorylation level of P65 and IκBα, which were elevated by IL-1β treatment, indicating that MLT can attenuate the IL-1β-induced activation of NF-κB signaling.

**Conclusion:**

The current evaluation showed that MLT can save IL-1β-impaired chondrogenesis of human BMSCs by restoring the pellet size and matrix accumulation, and maintaining the metabolic balance, reducing cell apoptosis. Our study also showed that MLT can attenuate the IL-1β-induced activation of the NF-κB signaling pathway, which is the most important pathway downstream of IL-1β, and plays a crucial role in inflammation, apoptosis, and metabolism. Thus, MLT has prospects for treating OA due to its multifaceted functions, such as mitigating inflammation, maintaining metabolic balance, and mitigating apoptosis.

## Background

Osteoarthritis (OA) is the most widespread degenerative arthritic disease across the globe, and it is a primary cause of disability, with radiographically determined OA impacting about 37% of the US population older than 60 years of age [1, 2]. Typical clinical characteristics include pain, joint dysfunction, and deformity, which all lower health-related quality of life. OA is anticipated to be the fourth-leading reason for disability by the year 2020 because of the aging of the world’s population [3]. This motivates us to further explore more OA treatment options, including stem-cell-based therapy.

OA has been thought of as a degenerative disease of the cartilage for a long time; however, increasing evidence suggests that inflammation plays a pivotal part in its pathogenesis. Inflammation takes part in the early course of OA, resulting in the metabolic dysfunction of chondrocytes, advancing the malfunction of articular cartilage, and eventually leads to the functional breakdown of synovial joints. Interleukin-1β (IL-1β) is a primary mediator of local inflammatory processes in OA [4, 5]. There are extensive studies that suggest elevated levels of IL-1β during the cartilage destruction cascade in the OA process [4, 6, 7]. IL-1β can change the differentiation and function of chondrocytes, which can then prompt the expression and activation of matrix metalloproteinases (MMPs) and a disintegrin and metalloproteinase with thrombospondin motifs (ADAMTS), enzymes that break down the cartilage matrix, encourage cell apoptosis, and are believed to be the downstream effectors of OA pathogenesis [4, 8–10]. Moreover, there is extensive literature that demonstrates the effects of IL-1β on chondrogenic MSCs [5, 11]. Wehling et al. [11] found that IL-1β inhibited chondrogenesis of MSCs in a dose-dependent manner and cell-based repair of lesions in articular cartilage will be compromised in inflamed joints.

Contemporary treatments for OA mainly focused on pain management, viscosupplementation, and joint replacement, which all simply target the clinical symptoms of the progressive stage of OA [7]. Drugs focused on fixing the injured cartilage due to OA are desperately required. Possible treatments, including anti-IL-1β, in OA animal models revealed lowered infiltration of inflammatory cells and cartilage injury [12]. Unfortunately, IL-1β blockade is linked to liver toxicity [13, 14]. Since articular cartilage has a limited self-repair capacity, the use of BMSCs to regenerate cartilage is an attractive approach due to the multiple differentiation abilities and the extensive resources of harvestable BMSCs available [15, 16]. MSCs, which inhabit bone marrow and numerous adult tissues, are able to self-renew and differentiate into various cell lineages, such as osteoblasts and chondrocytes. MSCs have been established in healthy and damaged cartilage and seem to keep at a minimum some promising ability to regenerate cartilage [17, 18]. Cartilage tissue-engineering repair strategies that depend on the chondrogenesis of MSCs are attractive, but in instances of OA they require chondrogenesis to take place within an inflamed environment. Moreover, MSCs interact with both the innate and adaptive immune systems, generally leading to abatement of ongoing inflammatory responses, which aggravates the damage caused by inflammatory factors like IL-1β [19]. However, there is surprisingly little in the literature concerning ways to stop or reverse IL-1β-induced impairment of chondrogenesis, which could greatly improve the clinical outcomes of cartilage tissue-engineering repair strategies for OA treatment [5, 11, 20].

Melatonin (MLT), best known as a modulator of circadian rhythms [21, 22], is reported to have multiple functions, including restriction of tumor development, immunomodulation, and antioxidation [23–27]. MLT and its metabolites modulate a variety of molecular signaling pathways including proliferation, apoptosis, metastasis, and inflammation, across a wide range of pathophysiological situations [28–30]. Further, MLT plays a pivotal part in managing skeleton establishment and growth. Our prior evaluation revealed that MLT can halt adipogenesis and encourage both osteogenic and chondrogenic differentiation of MSCs [31–33]. IL-1β is an important ligand of the NF-κB pathway, which is one of the most important pathways involved in inflammation and apoptosis. We believe that the NF-κB pathway plays a crucial role in IL-1β’s inhibitory effects in the process of chondrogenesis. With its powerful anti-inflammatory and prochondrogenic effects, we suggest that MLT could be a potential therapeutic compound for IL-1β-inhibited chondrogenesis by suppressing the activation of NF-κB signaling.

In the current evaluation, MLT was examined for its potential to encourage chondrogenic differentiation, retain metabolic balance, and lower cell apoptosis of human MSCs with the inflammatory factor IL-1β. MLT’s influence on the NF-κB pathway was also assessed. The objectives of this evaluation are to additionally establish the main part of MLT in the management of the differentiation of MSCs in a pathological environment and its potential underlying mechanism, offering additional evidence for the utilization of MLT in stem-cell-based OA treatment.

## Methods

### Antibodies and reagents

Recombinant human IL-1β was purchased from R&D (Minneapolis, MN, USA). MLT, Alcian blue solution, hydrochloride, EDTA, 1,9-dimethylmethylene blue (DMMB), and dye Hoechst 33,258 were purchased from Sigma-Aldrich (St. Louis, MO, USA). COL2A1 antibody was from Abcam (Cambridge, UK). The DAB Horseradish Peroxidase Color Development Kit was from (Beyotime Biotechnology, Beijing, China), and the MEBSTAIN Apoptosis TUNEL Kit Direct was from MBL International Co. (Woburn, MA, USA). The subsequent antibodies (Abs) were bought from Cell Signaling Technology (CST, Danvers, MA, USA): P65, phospho-P65, IκBα, phospho-IκBα, GAPDH, goat anti-rabbit IgG H&L (HRP), and goat anti-mouse IgG H&L (HRP).

### Separation and culture of MSCs

The study was authorized by the Ethical Committee of Sun Yat-sen University, and written informed consent was gained from each of the participants enrolled in the evaluation. MSCs were separated from bone marrow obtained from healthy volunteer donors as described previously [31, 33]. In short, the bone marrow specimens were diluted with PBS. Cells were then fractionated on a lymphoprep density gradient by centrifugation at 500 × *g* for 20 min. Interfacial mononuclear cells were gathered, resuspended in low-glucose Dulbecco’s modified Eagle medium (DMEM; Gibco, Waltham, MA, USA) augmented with 10% FBS (Gibco), and then seeded and incubated at 37 °C/5% CO_2_. After 48 h, nonadherent cells were eliminated by replacing the medium with fresh medium. The medium was then replaced every 3 days. When the cells approached 80–90% confluence, they were trypsinized, quantified, and plated again. Cells from passages 3–6 were utilized for the experiments.

### Chondrogenic differentiation

A high-density micromass culture system was used for the chondrogenic differentiation of human MSCs as described previously [33]. In short, MSCs were trypsinized, washed, and then resuspended at 2 × 10^7^ cells/ml in OriCell™ Human Mesenchymal Stem Cell Chondrogenic Differentiation Medium (Cyagen Biosciences Inc.). Droplets (12.5 μl) were carefully placed in each interior well of a 24-well plate. Cells were allowed to adhere at 37 °C for 2 h, followed by addition of 500 μl chondrogenic medium containing vehicle, 10 ng/ml IL-1β and vehicle (PBS with 0.1% BSA), or 10 ng/ml IL-1β and 50 nM MLT. The medium was replaced every 3 days and the pellets were harvested on days 7, 14, and 21.

### Real-time RT-PCR assay

Total RNA was removed with RNAiso Plus Reagent (Roche, Basel, Switzerland) and then changed to cDNA with PrimeScript™ RT Master Mix (Roche) based on the manufacturer’s instructions. Real-time PCR was conducted on a Light Cycler 480 Real-Time PCR Detection System (Roche) with SYBR Green I Master Mix (Roche). Expression levels were established for the following genes: *ACAN*, *COL2A1*, *COL10A1*, *SOX9*, *MMP9*, *MMP13*, and *ADAMTS4*. The expression level of the glyceraldehyde-3-phosphate dehydrogenase (*GAPDH*) gene acted as a reference. Every PCR was processed in triplicate. The Ct value of *GAPDH* was subtracted from the Ct value of the target gene (ΔCt), and the average ΔCt value of each replicate was documented. The relative expression levels of every gene were established with the 2^–ΔΔCt^ method. Primer sequences utilized in this evaluation are presented in Table 1.

**Table 1:** All primers for teal-time RT-PCR assay

Gene	Primer sequence
*COL2A1*	Sense: 5′-GGCAATAGCAGGTTCACGTACA-3′ Antisense: 5′-CGATAACAGTCTTGCCCCACTT-3′
*ACAN*	Sense: 5′-TGCATTCCACGAAGCTAACCTT-3′ Antisense: 5′-GACGCCTCGCCTTCTTGAA-3′
*SOX9*	Sense: 5′-AGCGAACGCACATCAAGAC-3′ Antisense: 5′-GCTGTAGTGTGGGAGGTTGAA-3′
*COL10A1*	Sense: 5′-CAAGGCACCATCTCCAGGAA-3′ Antisense: 5 ′-AAAGGGTATTTGTGGCAGCATATT-3′
*MMP9*	Sense: 5′-TGTACCGCTATGGTTACACTCG-3′ Antisense: 5 ′-GGCAGGGACAGTTGCTTCT-3′
*MMP13*	Sense: 5′-CCAGACTTCACGATGGCATTG-3′ Antisense: 5′-GGCATCTCCTCCATAATTTGGC-3′
*ADAMTS4*	Sense: 5′-GAGGAGGAGATCGTGTTTCCA-3′ Antisense: 5 ′-CCAGCTCTAGTAGCAGCGTC-3′
*GAPDH*	Sense: 5′-AGAAAAACCTGCCAAATATGATGAC-3′ Antisense: 5 ′-TGGGTGTCGCTGTTGAAGTC-3′

### Alcian blue staining

Micromasses were fixed in 4% paraformaldehyde for 3 h and then dehydrated with ethanol, washed with xylene, and embedded in paraffin. Sections with a thickness of 4 μm were cut and coated on the glass slides. We then deparaffinized the slides and hydrated them three times with distilled water, and Alcian blue solution (pH 2.5; Sigma-Aldrich) was added and incubated for 1 h at room temperature. After a removal of staining reagents, the slides were washed in running tap water for 2 min. Then mount with resinous mounting medium. Finally, the sections were photographed with an Olympus BX51 microscope (Olympus, Tokyo, Japan).

### Quantitative analysis of glycosaminoglycan

Pellets were cleaned and digested in PBS with 0.03% papain (Merck, Darmstadt, Germany), 5 mM cysteine hydrochloride, and 10 mM EDTA for 16 h at 65 °C. The glycosaminoglycan (GAG) concentration was quantified with a 1,9-dimethylmethylene blue dye binding assay. In short, a portion of the lysate was reacted with DMMB solution for 10 min, and the absorbance at 525 nm was established with Varioskan Flash (Thermo Scientific, Waltham, MA, USA). Pellet digests were taken through three freeze–thaw cycles, and aliquots were added to 100 ng/ml of Hoechst Dye 33,258 (Sigma) in 10 mM Tris (pH 7.4), 1 mM disodium EDTA, and 100 mM NaCl. DNA concentration was determined by fluorescent dye Hoechst 33,258 binding assay with a SpectraMax M5 microplate reader (Molecular Devices, Sunnyvale, CA, USA). Fluorescence was measured using excitation and emission wavelengths of 485 nm and 528 nm, respectively, and DNA concentrations were determined relative to a lambda DNA standard curve. For GAG synthetic activity, the resulting GAG amounts were normalized to the amount of DNA for each sample.

### Immunohistochemical analysis

We used 4% paraformaldehyde for fixation of the tissues at room temperature for 1 h. Paraffin sections (4 μm thick) were prepared and immunohistochemical (IHC) analysis was performed using a Histostain-Plus Kit (Thermo Fisher Scientific, Waltham, MA, USA). We used 5% bovine serum albumin as the blocking reagent. The specimen was treated for 30 min at room temperature, and the tissues were incubated with the anti-COL2A1 antibody (1:500) at 4 °C overnight. Detection was performed with a DAB Horseradish Peroxidase Color Development Kit (Origin Technologies, Inc.).

### Terminal deoxynucleotidyl-transferase-mediated dUTP nick end labeling assay

A terminal deoxynucleotidyl-transferase-mediated dUTP nick end labeling (TUNEL) assay was conducted with a MEBSTAIN Apoptosis TUNEL kit direct (MBL International Co.) based on the manufacturer’s instructions. The percentage of TUNEL-positive cells relative to propidium iodide (PI)-stained cells was calculated. Three independent experiments were conducted and quantified for each experimental group.

### Immunoblotting analysis

Pellets were cleaned three times with cold PBS and gathered in RIPA (Beyotime, Shanghai, China), adding 1% protease inhibitor and phosphatase inhibitor. Pellets were exposed to the liquid nitrogen for 15 min and pellet lysates were obtained using a TissueLyser (QIAGEN, Germany). Identical portions of each specimen were subjected to SDS-polyacrylamide gel electrophoresis (PAGE) and moved to PVDF transfer membranes (Millipore). Membranes were halted with 5% nonfat milk for 1 h at room temperature and then incubated with the anti-P65, anti-phospho-P65, anti-IκBα, and anti-phospho-IκBα specified antibodies (1:1000) at 4 °C overnight. Antibody-specific labeling was noted by incubation with secondary antibodies (1:2000) for 1 h at room temperature and observed with an ECL kit (Millipore). The band was established with ImageJ software and normalized to *GAPDH* (1:2000) as the loading control.

### Statistical analysis

Comparisons of perimeters, GAG content, gene expressions, and quantitation of protein expressions were performed using a two-tailed independent Student’s *t* test. Statistical analyses for comparisons of apoptosis rate and relative protein expressions were performed using chi-square or Fisher’s exact tests when appropriate. All statistical analyses were conducted with SPSS 20.0 statistical software (SPSS, Chicago, IL, USA) and GraphPad Prism 5.01. The level of statistical significance was established at *P* < 0.05.

## Results

**Fig. 1:**
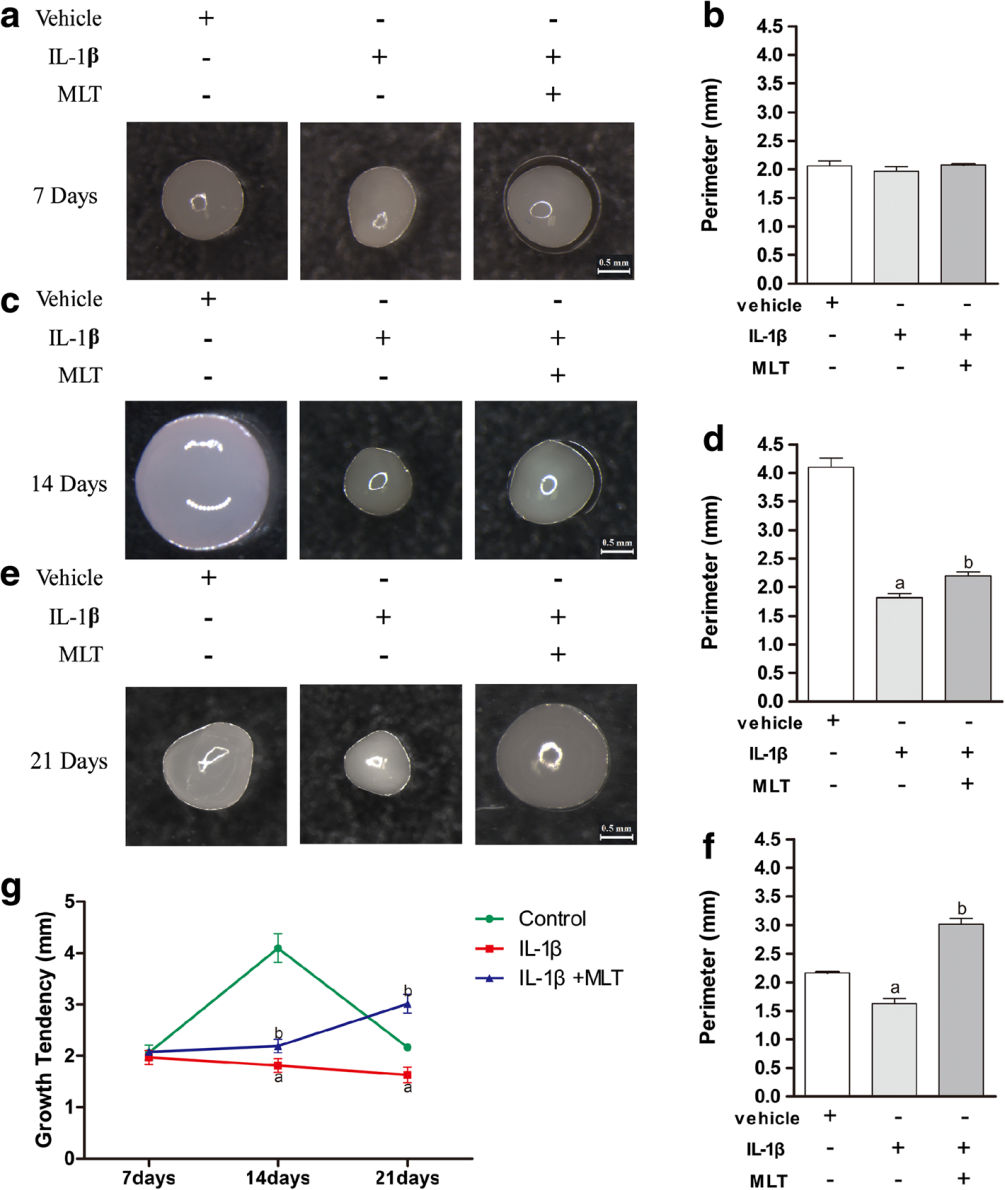
Impact of IL-1β and MLT on cartilage pellet development throughout chondrogenesis of human mesenchymal stem cells (hMSCs). hMSCs prompted into chondrogenesis in chondrogenic medium with vehicle, 10 ng/ml IL-1β and vehicle, or 10 ng/ml IL-1β and 50 nM MLT via high-density micromass culture system. Cartilage pellets cultured for 7 (**a**), 14 (**c**), and 21 (**e**) days and observed by stereoscopic microscope in representative samples. Scale bars: 0.5 mm. Perimeters of pellets cultured for 7 (**b**), 14 (**d**) and 21 (**f**) days of chondrogenesis , 12 pellets used for measurement in each group. **g** Growth tendency of pellets from day 7 to day 21 in different groups. ^a^*P* < 0.05 versus control group; ^b^*P* < 0.05 versus IL-1β group. IL-1β interleukin-1β, MLT melatonin

To examine the impacts of MLT on chondrogenic differentiation of MSCs with IL-1β, chondrogenic differentiation was prompted in human MSCs in chondrogenic medium with vehicle, 10 ng/ml IL-1β and vehicle, or 10 ng/ml IL-1β and 50 nM MLT. No difference was discovered between groups in pellet size on day 7 of chondrogenesis (Fig. 1a); quantitative analysis of the perimeters of the pellets also confirmed that there was no difference in chondrogenesis on day 7 between different groups (Fig. 1b). However, the cartilage pellets treated with IL-1β were smaller than those of the controls in the 14-day and 21-day groups and additional MLT treatment partially restored the size of the pellets (Fig. 1cf–f).

**Fig. 2:**
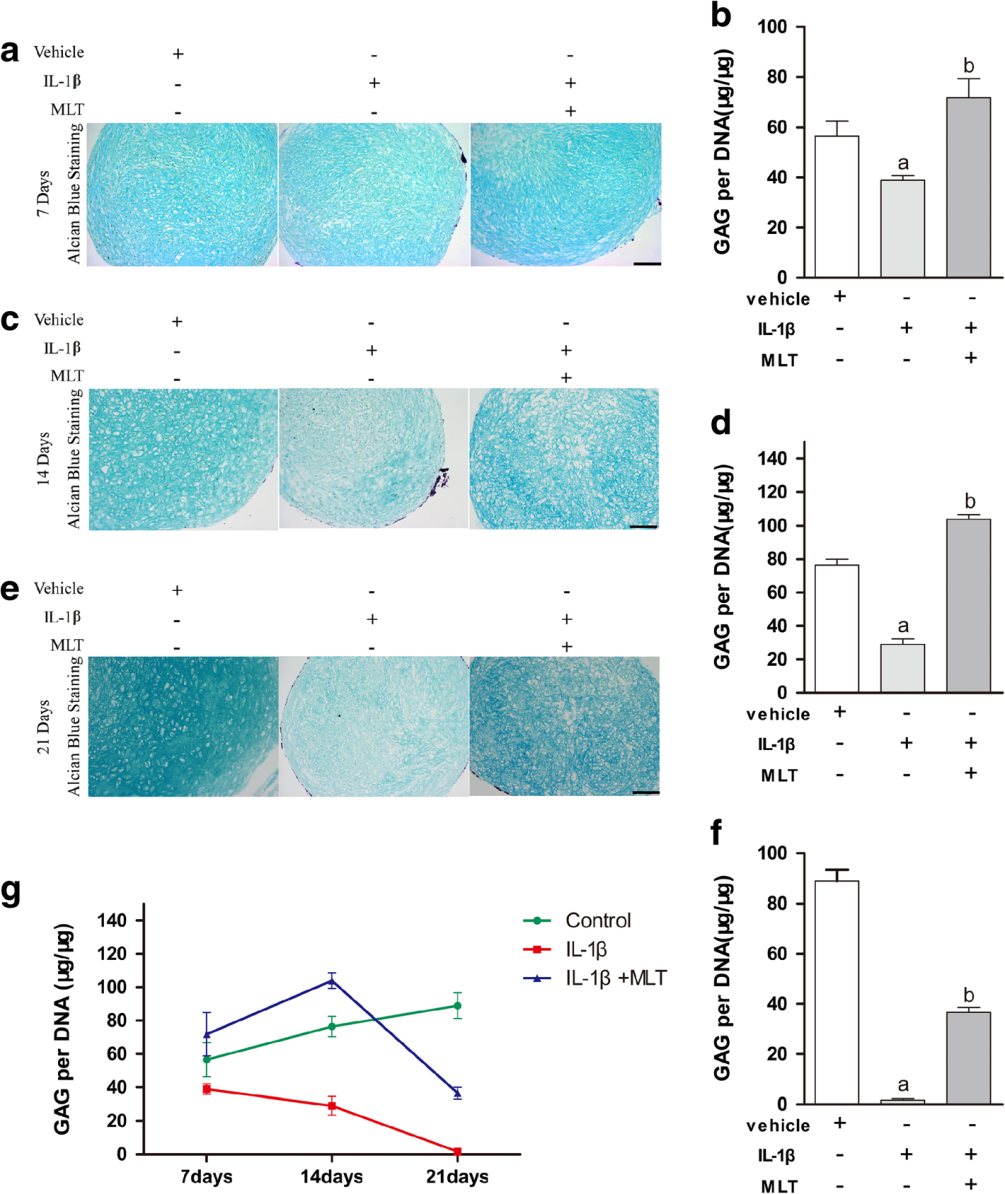
MLT rescued IL-1β-impaired matrix accumulation during chondrogenesis process. Alcian blue staining performed to visualize matrix accumulation. Pellets of different stages harvested. Paraformaldehyde 4% used for fixation. Paraffin sections (4 μm thick) prepared for staining. Cartilage pellets cultured for 7 (**a**), 14 (**c**), and 21(**e**) days observed by microscope in representative samples. Scale bars: 0.1 mm. Quantitative analysis of glycosaminoglycan (GAG) evaluated typical matrix accumulation during chondrogenesis. GAG content quantitatively examined and normalized by DNA content on days 7 (**b**), 14 (**d**), and 21 (**f**). Outcomes representative of three separate experiments. **g** Changing pattern of GAG/DNA from day 7 to day 21 in different groups. ^a^*P* < 0.05 versus control group; ^b^*P* < 0.05 versus IL-1β group. IL-1β interleukin-1β, MLT melatonin

Alcian blue staining was used for evaluation of cartilage matrix synthesis and accumulation. The results of Alcian blue staining and the quantitative analysis of glycosaminoglycan (GAG) showed that GAG synthesis and matrix deposition decreased in the presence of IL-1β and was elevated by MLT treatment on day 7 (Fig. 2a, b), day 14 (Fig. 2c, d), and day 21 (Fig. 2e, f) (*P* < 0.05). These outcomes revealed that IL-1β suppresses the accumulation of matrix during chondrogenesis of MSCs, and MLT can rescue the impacts of IL-1β.

**Fig. 3:**
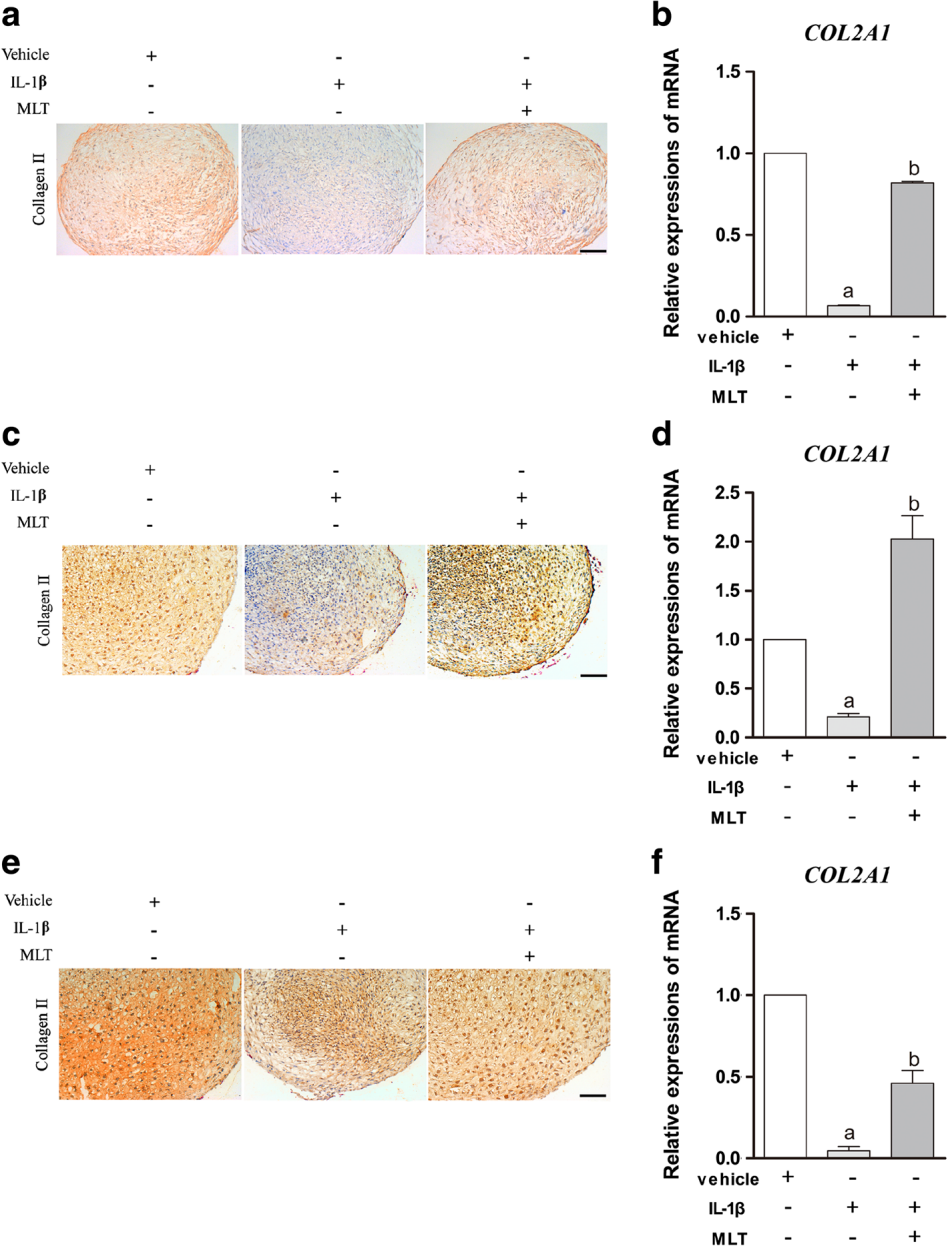
MLT reversed IL-1β-inhibited COL2A1 expression at both mRNA and protein levels. qPCR and IHC analysis assessed COL2A1 expressions at 7, 14 and 21 days. mRNA expression of *COL2A1* measured by qPCR and normalized to *GAPDH*. Relative expression levels of gene on 7 (**b**), 14 (**d**), and 21 (**f**) days representative of three independent experiments. ^a^*P* < 0.05 versus control group; ^b^*P* < 0.05 versus IL-1β group. IHC staining of collagen type II performed after 7 (**a**), 14 (**c**), and 21 (**e**) days of differentiation. Scale bars: 0.1 mm. IL-1β interleukin-1β, MLT melatonin

To further confirm the effects of IL-1β and MLT on the process of chondrogenesis, the level of expression of the typical chondrogenic marker *COL2A1* was detected using real-time RT-PCR and immunohistochemical (IHC) staining. These findings revealed that IL-1β dramatically inhibited collagen II expression on day 7 (Fig. 3a, b), day 14 (Fig. 3c, d), and day 21 (Fig. 3e, f) during chondrogenesis, while the additional MLT treatment reversed this situation at both mRNA and protein levels (*P* < 0.05). These results showed that IL-1β suppresses gene and protein expression of typical chondrogenic marker *COL2A1* of chondrogenic MSCs. Again, MLT can counterbalance the effects of IL-1β.

**Fig. 4:**
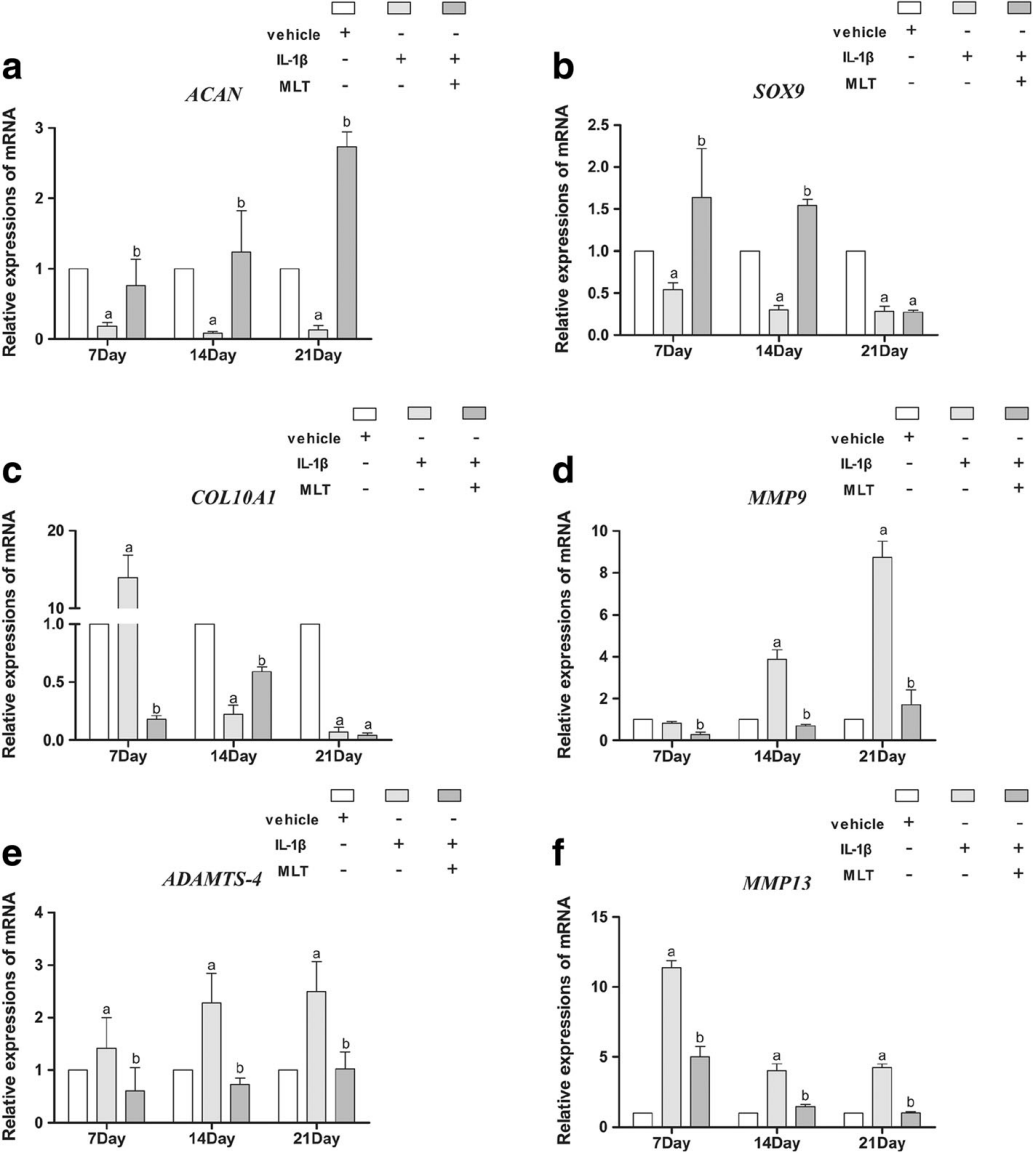
IL-1β and MLT treatment affected chondrogenic and apoptotic marker expression at mRNA level. Expression of *ACAN* (**a**), *SOX9* (**b**), *COL10A1* (**c**), *MMP9* (**d**), *MMP13* (**e**), and *ADAMTS4* (**f**) measured by qPCR and normalized to *GAPDH*. Relative expression level of each gene on different stages of differentiation representative of three independent experiments. ^a^*P* < 0.05 versus control group; ^b^*P* < 0.05 versus IL-1β group. IL-1β interleukin-1β, MLT melatonin

Next, RT-PCR was utilized to explore the impacts of IL-1β and MLT on the expressions of other chondrogenic markers, such as *ACAN*, *COL10A1*, and *SOX9.* As shown in Fig. 4a, b, the level of expression of *ACAN* and *SOX9* are consistent with the pattern of expression of *COL2A1*. IL-1β downregulated *ACAN* and *SOX9* expression, whereas after the addition of MLT to IL-1β,  upregulation of those genes was were observed on days 7 and 14 (*P* < 0.05). Of note, on day 21 the effects of IL-1β and MLT on *ACAN* were consistent with those on days 7 and 14 (*P* < 0.05), while the effect of MLT on *SOX9* was gone. *COL10A1*, a chondrogenic and a hypertrophic marker, was found to be elevated by IL-1β treatment and then declined after the addition of MLT on day 7 (Fig. 4c) (*P* < 0.05). IL-1β downregulated *COL10A1* expression, whereas MLT reversed the effect on day 14. The effect of MLT on *COL10A1* was also gone on day 21. We also explored the impacts of IL-1β and MLT on catabolic and proapoptotic markers like *MMP9*, *MMP13*, and *ADAMTS4*. On day 7, IL-1β treatment had little effect on *MMP9* expression, while MLT treatment decreased the expression of *MMP9*; IL-1β then upregulated *MMP9* expression on days 14 and 21, while MLT downregulated the level of expression of *MMP9* (Fig. 4d) (*P* < 0.05). IL-1β upregulated *MMP13* and *ADAMTS4* expression, whereas the addition of MLT to IL-1β then downregulated their expression on days 7, 14, and 21 (Fig. 4e, f) (*P* < 0.05). The impacts of IL-1β and MLT on these proapoptotic markers suggested that MLT could have a part in the chondrogenesis process as an anticatabolic and antiapoptotic agent.

**Fig. 5:**
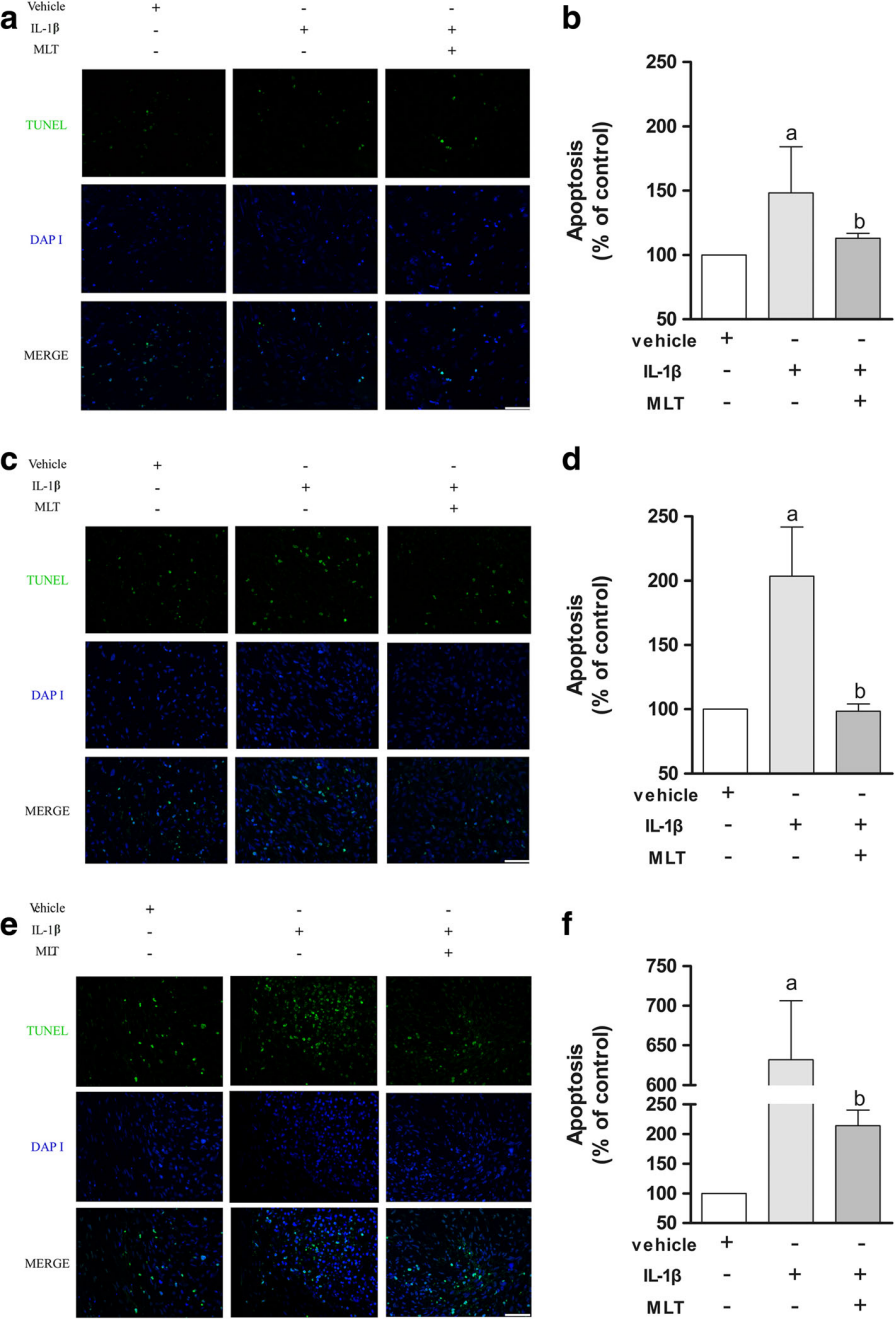
MLT protected human MSCs from IL-1β-induced apoptosis in process of chondrogenesis. TUNEL assay showed apoptotic MSCs (green fluorescence) with PI-labeled nuclei (blue fluorescence) on days 7 (**a**), 14 (**c**), and 21 (**e**). Positive rates statistically analyzed on right correspondingly (**b**, **d**, **f**), and data represent mean ± SD of three independent experiments. Scale bars = 50 μm. ^a^*P* < 0.05 versus control group; ^b^*P* < 0.05 versus IL-1β group. DAPI 4′,6-diamidino-2-phenylindole, IL-1β interleukin-1β, MLT melatonin, TUNEL terminal deoxynucleotidyl-transferase-mediated dUTP nick end labeling

We performed a TUNEL assay to determine how IL-1β and MLT influenced the cell fate of chondrogenic MSCs. IL-1β treatment increased the percentage of TUNEL-positive cells compared to control, and the addition of MLT significantly reversed this effect mainly on day 7 (Fig. 5a, b), day 14 (Fig. 5c, d), and day 21 (Fig. 5e, f) (*P* < 0.05). These results confirmed that IL-1β induces MSC apoptosis during the process of chondrogenesis and MLT plays the role of an antiapoptotic agent, reducing MSC apoptosis and rescuing IL-1β-impaired chondrogenesis.

**Fig. 6:**
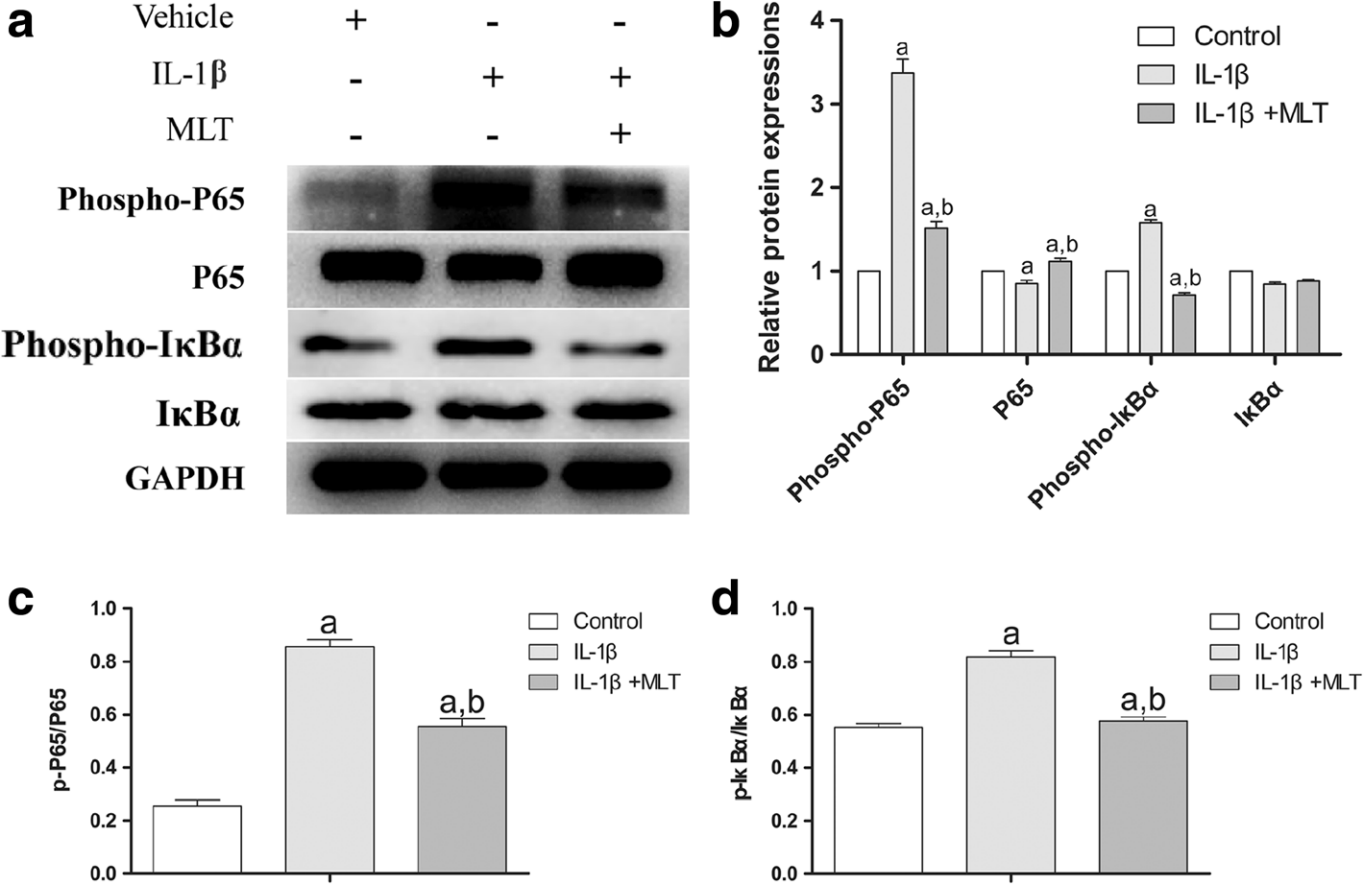
MLT downregulated IL-1β-enhanced phosphorylation of P65 and IκBα in NF-κB pathway. Immunoblotting (**a**) conducted to detect expression and total and phosphorylated P65 and IκBα to reflect activation of NF-κB signaling. Phospho-P65, P65, phospho-IκBα, and IκBα protein levels established (**b**). Ratio of relative protein expression of p-P65 to relative protein expression of P65 (p-P65/P65) (**c**), and ratio of relative protein expression of p-IκBα to relative protein expression of IκBα (p-IκBα/IκBα) examined (**d**). ^a^*P* < 0.05 versus control group; ^b^*P* < 0.05 versus IL-1β group. IL-1β interleukin-1β, MLT melatonin

The NF-κB pathway is one of the most crucial pathways in apoptosis, and IL-1β is a pivotal ligand of the NF-κB pathway. We believe that the NF-κB pathway plays a crucial part in IL-1β’s inhibitory effects in chondrogenesis. To test this hypothesis, we determined the levels of expression and activity of key molecules of the NF-κB pathway of the pellets on day 21 using immunoblotting. As shown in Fig. 6a, both IL-1β and MLT regulated the phosphorylation levels of p-P65 and p-IκBα. While the expression of total P65 was downregulated by IL-1β, MLT then elevated the P65 level (*P* < 0.05). Total IκBα remained the same in different groups (Fig. 6b); however, IL-1β upregulated the phosphorylation levels of P65 and IκBα, causing increased NF-κB activation. In contrast, MLT downregulated the phosphorylation levels of P65 and IκBα, thus attenuating NF-κB activation (Fig. 6c, d) (*P* < 0.05). These results indicated that MLT attenuated IL-1β’s impact on the NF-κB signaling pathway.

## Discussion

MSCs have already been intensively examined and utilized in clinical trials for regenerative therapies in the skeletal system [17, 34]. Recent studies demonstrate that MSCs may act as anti-inflammatory agent to allow the joint to self-repair; MSCs combined with appropriate scaffolds can form cartilaginous or even osseous compartments to repair cartilage [35]. However, such therapies have not been successful thus far and they did not have the intended impact [34]. A large issue that researchers encounter is the inflamed surroundings [5, 7, 11]. Our intention is to use the in-vitro chondrogenesis model to test the possible effect of MLT on IL-1β-impaired cartilage formation and the underlying mechanism. We want to prove that MLT can act as a possible drug that helps to optimize the cartilage tissue engineering system or even a drug that helps the cartilage to self-repair under inflammatory conditions. To the best of our knowledge, our evaluation is the first to discover that MLT can function as a proanabolic, anticatabolic, and antiapoptotic agent for MSCs in the chondrogenic process under IL-1β challenge.

The majority of previous studies [5, 11] that assessed either the effect of inflammatory cytokines or MLT have been based on a single point in time, which disregards valuable information in the whole process of chondrogenesis. Liu et al. [5] investigated the role of MLT on proinflammatory cytokine-inhibited chondrogenesis in synovium mesenchymal stem cells and the possible role of reactive oxygen species in its process. They found that the chondroprotective effect of MLT was potentially correlated to decreased ROS, preserved SOD, and suppressed levels of MMPs. However, in the current study, we used BMSCs induced for 7, 14, and 21 days to help monitor the chondrogenic process in a continuous way, through which we found that on day 7 IL-1β and MLT barely affected the chondrogenesis in pellet size and matrix accumulation. Instead, on day 21 the most obvious effect of IL-1β and MLT was observed among the different stages. Specifically, on day 7 the induced cartilage pellets showed no difference in size or perimeter or Alcian blue staining among different groups, while the GAG content, *COL2A1*, *ACAN*, and *SOX9* expression was altered by IL-1β and MLT treatment. Of note, the mRNA level of *COL10A1*, which is a cartilage hypertrophic marker, was surprisingly upregulated in the IL-1β group. Surprisingly, MLT downregulated the expression level of *COL10A1*. Since hypertrophy is the terminal stage of cartilage before apoptosis, this result would lead us to realize that apoptosis of induced MSCs may be partially responsible for unsatisfactory outcomes of chondrogenesis in an inflamed environment. On days 14 and 21, pellets treated with IL-1β showed smaller size and perimeter, decreased matrix accumulation, and downregulation of typical chondrogenic marker *COL2A1* at both mRNA and protein levels, whereas MLT restored the effects. *ACAN* shared the same changing patterns with *COL2A1* only on day 14. *SOX9*, a marker of cartilage formation, remained at low levels in the IL-1β and MLT groups, suggesting 21 days is a terminal stage of chondrogenesis in the presence of IL-1β. However, *MMP9*, *MMP13*, and *ADAMTS4*, known as catabolic and proapoptotic markers, were upregulated across all three stages in the IL-1β group, and addition of MLT reversed the expression levels of the three genes. Lastly, the TUNEL assay confirmed the hypothesis that MLT plays a role in rescuing IL-1β-impaired chondrogenesis as an antiapoptotic agent.

Our study has some limitations. Firstly, IL-1β is a highly crucial inflammatory factor, and it has a pivotal part in the pathogenesis of OA. Apart from IL-1β, numerous soluble inflammatory mediators (TNF-α, IL-6, etc.) have been determined to be present in OA joint tissues and fluids [7, 36]. However, Liu et al. [5] demonstrated that IL-1β and TNF-α had an inhibitory impact on the chondrogenesis of MSCs. IL-1β was found to have a more potent effect than TNF-α. Thus, we chose IL-1β as representative to create an inflamed environment for MSCs. Secondly, our study lacks evidence from the protein level. MMPs and collagens are important proteins evolving in the cartilage metabolism. We use *MMP9*/*MMP13*/*COL10A1* gene expression as catabolic, proapoptotic, and hypertrophic markers. We will continue our work to explore the protein expression and functions of MMPs and ColX. Thirdly, we did not fully excavate the underlying mechanisms of MLT’s effect. Guo et al. [37] demonstrate that MLT inhibits Sirt1-dependent NAMPT and NFAT5 signaling in chondrocytes to attenuate OA. However, in the chondrogenic process with IL-1β presence, MLT’s role is still not clear. Apoptosis signaling pathways in OA and the potential protective part of MLT are well established [38]. The NF-κB signaling pathway is crucial in the apoptosis of chondrocytes. IL-1β is a classical ligand of the NF-κB pathway. To test this hypothesis, we detected the expression level and activity of key molecules of the NF-κB pathway. The results showed that on day 21, IL-1β upregulated the phosphorylation levels but not the expression of P65 and IκBα, causing increased NF-κB activation. In contrast, MLT downregulated the phosphorylation of P65 and IκBα, thus attenuating NF-κB signaling activation. These outcomes suggested that MLT diminished IL-1β-induced stimulation of the NF-κB signaling pathway. Moreover, further study is needed to explore the exact mechanism of how MLT regulates NF-κB and also the possible mechanisms that involve different pathways in the process. Furthermore, the growth tendency of chondrogenic pellets in the control group shrank from day 14 to day 21, which is not consistent with published results. We speculated that this was due to different cell sources and cell types and different methods to induce chondrogenesis. Yang et al. [39] used human adipose-derived stem cells and a pellet culture system to induce chondrogenesis, their results showing that the pellets in the control groups were largest on day 7, and shrank from day 7 to days 14 and 21. We found in our own results that the pellets on day 14 had the most hyaline cartilage characteristics; on day 21 the pellets showed a hypertrophic cartilage phenotype, which may explain the reason why the pellets shrank. Lastly, our results were based on in-vitro data only, which limited the evidence level. We are constructing an OA rat model to testify the role of MLT, the results (data not shown) showing that MLT treatment can repair the cartilage damage caused by OA.

## Conclusion

The current evaluation offers evidence that further MLT treatment can save the IL-1β-impaired chondrogenesis of MSCs in various ways including pellet size, glycosaminoglycan accumulation, *COL2A1* expression at mRNA and protein levels, and *ACAN*, *SOX9*, and *COL10A1* expression levels. Moreover, MLT may achieve this through rescuing the increased apoptosis of IL-1β-treated MSCs and the elevated expression of *MMP9*, *MMP13*, and *ADAMTS4* in the differentiating process. MLT may have rescued IL-1β-impaired chondrogenesis of MSCs by affecting the NF-κB pathway. Additional evaluations have focused on unraveling the particular mechanisms through which MLT diminished IL-1β-prompted apoptosis in MSCs and verified the therapeutic value of MLT in stem-cell-based therapies for OA.
